# Dibutyltin Compounds Effects on PPARγ/RXRα Activity, Adipogenesis, and Inflammation in Mammalians Cells

**DOI:** 10.3389/fphar.2017.00507

**Published:** 2017-08-02

**Authors:** Flora A. Milton, Mariella G. Lacerda, Simone B. P. Sinoti, Pedro G. Mesquita, Dileesh Prakasan, Michella S. Coelho, Caroline L. de Lima, Alexandre G. Martini, Gabriela T. Pazzine, Maria de F. Borin, Angelica A. Amato, Francisco de A. R. Neves

**Affiliations:** ^1^Laboratory of Molecular Pharmacology, Faculty of Health Science, University of Brasilia Brasilia, Brazil; ^2^Department of Basic Science Education, Federal Fluminense University Niterói, Brazil

**Keywords:** tributyltin, dibutyltin, peroxisome proliferator activated receptor gamma, adipogenesis, proinflammatory genes

## Abstract

Organotins are a group of chemical compounds that have a tin atom covalently bound to one or more organic groups. The best-studied organotin is tributyltin chloride, which is an environmental pollutant and an endocrine disruptor. Tributyltin chloride has been shown to bind to PPARγ/RXRα and induces adipogenesis in different mammalian cells. However, there are few studies with other organotin compounds, such as dibutyltins. The aim of this study was to investigate the effect of dibutyltins diacetate, dichloride, dilaurate, and maleate on the transcriptional activity of the nuclear PPARγ and RXRα receptors, and on adipogenesis and inflammation. Analogous to tributyltin chloride, in reporter gene assay using HeLa cells, we observed that dibutyltins diacetate, dichloride, dilaurate, and maleate are partial agonists of PPARγ. Unlike tributyltin chloride, which is a full agonist of RXRα, dibutyltins dichloride and dilaurate are partial RXRα agonists. Additionally, the introduction of the C285S mutation, which disrupts tributyltin chloride binding to PPARγ, abrogated the dibutyltin agonistic activity. In 3T3-L1 preadipocytes, all dibutyltin induced adipogenesis, although the effect was less pronounced than that of rosiglitazone and tributyltin chloride. This adipogenic effect was confirmed by the expression of adipogenic markers Fabp4, Adipoq, and Glut4. Exposure of 3T3-L1 cells with dibutyltin in the presence of T0070907, a specific PPARγ antagonist, reduced fat accumulation, suggesting that adipogenic effect occurs through PPARγ. Furthermore, dibutyltins dichloride, dilaurate, and maleate inhibited the expression of proinflammatory genes in 3T3-L1 cells, such as Vcam1, Dcn, Fn1, S100a8, and Lgals9. Additionally, in RAW 264.7 macrophages, tributyltin chloride and dibutyltin dilaurate reduced LPS-stimulated TNFα expression. Our findings indicate that dibutyltins diacetate, dichloride, dilaurate, and maleate are PPARγ partial agonists and that dibutyltins dichloride and dilaurate are also partial RXRα agonists. Furthermore, dibutyltins induce adipogenesis in a PPARγ-dependent manner and repress inflammatory genes in 3T3-L1 and RAW 264.7 cells. Although dibutyltins display some partial PPARγ/RXRα agonistic effects, the translation of cell-based results assays into in vivo effects on inflammation and insulin resistance is not entirely known. Nevertheless, further studies are necessary to address their effects in different periods of life and to elucidate the actions of organostanic compounds in whole-body context.

## Introduction

Organotins comprise a group of chemical compounds that have a tin atom covalently bound to one or more organic groups (Hoch, 2001). The first organotin was commercially registered in 1936 as a stabilizer for synthetic polymers (Fent, 1996). However, in the 1950s, the discovery of their biocidal properties expanded the range of applications and boosted the production of organotins (Fent, 1996).

The best-studied organotin is tributyltin chloride. Its use became widespread as antifouling paint on boats, ships and shipyards in the 1970s and 1980s (Fent, 1996), leading to contamination of water and sediments (Maguire et al., 1982), as well as of several species of aquatic life (Maguire et al., 1982; Alzieu and Heral, 1984; Alzieu et al., 1986; Iwata et al., 1994). In many marine species, exposure to tributyltin chloride causes imposex, the abnormal induction of male characteristics in females (Evans and Nicholson, 2000; McAllister and Kime, 2003). Although prohibited as antifouling paint from 2008 (Brown, 2000; Gipperth, 2009), tributyltin chloride is still used in industrial processes, as catalyst and stabilizer in various chemical reactions [(BAuA [Federal Institute for Occupational Safety and Health], 2013)].

Besides imposex, which is due to aromatase inhibition (McAllister and Kime, 2003), tributyltin chloride also has endocrine disrupting effects on adipogenesis, through its action on nuclear receptors PPARγ and RXRα (Grün et al., 2006; le Maire et al., 2009). Adipogenesis induced by PPARγ and RXRα ligands was demonstrated in cell lineages both committed and uncommitted to adipocyte differentiation (Kanayama et al., 2005; Grün et al., 2006; Kirchner et al., 2010), as well as in *in vivo* models (Grün et al., 2006; Zuo et al., 2009).

Dibutyltin is another organotin environmental pollutant, of which 18 types are registered by the European Chemicals Agency (ECHA) (European Chemicals Agency [ECHA], 2016). They are used in the manufacture of various products containing plastic and rubber materials, such as food packaging, toys, shoes, telephones, and tires, in reagents for treating paper, wood, leather, and fur, in the treatment of metallic and non-metallic surfaces, in pH regulators, and in water treatment products (Risk and Policy Analysts Ltd. [RPA], 2007; Environment and Climate Change Canada [ECCC], 2009; European Chemicals Agency [ECHA], 2016). Data from the European Tin Catalysts Association (ETICA), from 2007, indicate that about 90% of organotins produced up to that time were in the form of mono or dibutyltin (Risk and Policy Analysts Ltd. [RPA], 2007). However, unlike tributyltin chloride, few studies addressed the biological effects of dibutyltin compounds exposure, and when performed they were mainly focused on dibutyltin dichloride.

Based on studies of dibutyltin dichloride, ECHA classifies the different types of dibutyltins as toxic for reproduction (European Chemicals Agency [ECHA], 2016). In 2014, the TIB Chemicals company filed a proposal with ECHA to revise this classification for dibutyltin dilaurate, arguing that the studies on which it is based are old and probably had contamination by tributyltin chloride (TIB Chemicals AG, 2014). Regarding adipogenesis, a 2011 study in BMS2 murine multipotent stromal cells showed that dibutyltin dichloride induced adipocyte differentiation, albeit with less efficacy than tributyltin chloride (Yanik et al., 2011), yet the adipogenic mechanism underlying it was not investigated. Furthermore, the adipogenic effects of other dibutyltin compounds such as dibutyltin diacetate, dibutyltin dilaurate, and dibutyltin maleate were not previously evaluated.

Since PPARγ is the master regulator of adipocyte differentiation (Lehrke and Lazar, 2005), and both dibutyltin dichloride (Yanik et al., 2011) and tributyltin chloride promote adipogenesis on cell cultures, the latter through PPARγ/RXRα activation (Kanayama et al., 2005; Grün et al., 2006; Kirchner et al., 2010), it is presumable that the adipogenic effects of dibutyltins on cell cultures are mediated by PPARγ and/or RXRα signaling pathway. Moreover, PPARγ activation is also linked to anti-inflammatory effects (Lehrke and Lazar, 2005). Aiming to further understand the actions of dibutyltins, we investigated the effects of four of these compounds – dibutyltin diacetate, dibutyltin dichloride, dibutyltin dilaurate, and dibutyltin maleate – on the transcriptional activity of PPARγ and RXRα, adipogenesis in 3T3-L1 preadipocytes and the expression of inflammatory response-related genes in 3T3-L1 adipocytes and RAW 264.7 macrophages.

## Materials and Methods

### Transactivation Assay and Reporter Gene

Human cervical cancer HeLa cells (4 × 10^4^ cells/well, in 48-well plate), from the Cell Bank of Rio de Janeiro, Brazil, were co-transfected with expression vectors containing cDNA for chimeric nuclear receptors, including the ligand binding domain (LBD) of RXRα, PPARγ, or PPARγ with C285S mutation fused to the DNA binding domain (DBD) of the yeast transcription factor GAL4, and with a plasmid containing the luciferase reporter gene, kindly provided by Dr. Paul Webb from Methodist Research Institute, TX, United States. Transfections were performed with Lipofectamine (Lipofectamine 2000 Transfection Reagent) according to the manufacturer’s instructions. Cells were exposed for 24 h to vehicle (DMSO), 10 μM rosiglitazone (Cayman Chemical), 10 μM 9-*cis*-retinoic acid (Cayman Chemical), and tributyltin chloride, dibutyltin diacetate, dibutyltin dichloride, dibutyltin dilaurate, or dibutyltin maleate, at concentrations ranging from 0.00001 μM to the maximum non-toxic concentration determined in cell viability assays, performed as previously described (Mosmann, 1983) (data not shown). The maximum non-toxic concentrations of each of organotin compounds were 0.1 μM for tributyltin chloride, 1 μM for dibutyltin diacetate, 1 μM for dibutyltin dichloride, 10 μM for dibutyltin dilaurate, and 0.1 μM for dibutyltin maleate. The organotins were purchased from Sigma–Aldrich, and their purity ranged from 95 to 96%.

Luciferase activity was measured in a luminometer with a luciferase reporter assay kit (Promega) according to the manufacturer’s instructions. Results were reported as luciferase activity induced by the different compounds relative to vehicle (DMSO). Each experiment was performed in triplicate and repeated at least three times. The effect of different concentrations was compared using analysis of variance (ANOVA) followed by multiple comparisons of Bonferroni test (GraphPad Prism Software, version 5.01). The significance criterion for all analyses was *p* < 0.05.

### Adipocyte Differentiation Assay

In order to evaluate the dibutyltins effects on adipogenesis, 3T3-L1 were used, a cell line derived from the embryonic tissue of Swiss mice, kindly provided by Dr. Keith Yamamoto (University of California, San Francisco, CA, United States). 3T3-L1 preadipocytes were cultured in 6-well plates (48 × 10^3^ cells/well) and maintained at 37°C and 5% CO_2_ in DMEM medium with 10% fetal calf serum. Two days after confluence, preadipocytes were induced to differentiate into adipocytes with 10 μg/mL human insulin (Sigma–Aldrich) in DMEM with 10% fetal bovine serum. After another 48 h, cells were maintained with 5 μg/mL human insulin in DMEM plus 10% fetal bovine serum, that was replaced every 48 h, until fixation or cell harvesting. During all the differentiation period, cells were exposed to vehicle (DMSO), 0.1 μM rosiglitazone, or with organotin compounds, which were added to the culture medium containing insulin. The exposition was performed every 48 hours, always starting at the moment of differentiation induction (2 days after cell confluence). Preadipocytes were also differentiated in the presence or absence of the PPARγ antagonist T0070907 (1 μM) (Cayman Chemical), added to the culture medium every 12 h. Adipocyte differentiation assays were performed based on the protocol described in 3T3-L1 Cell Care Manual: Maintenance and Differentiation of 3T3-L1 Preadipocytes to Adipocytes (ZenBio, 2015).

For the assessment of intracellular lipid accumulation, preadipocytes were differentiated as described above, in the presence or absence of the PPARγ antagonist T0070907 (1 μM), in addition to vehicle (DMSO), 0.1 μM rosiglitazone, or tributyltin chloride, dibutyltin diacetate, dibutyltin dichloride, dibutyltin dilaurate or dibutyltin maleate at concentrations ranging from 0.00001 μM to the maximum non-toxic concentration determined in cell viability assays, performed as previously described (Mosmann, 1983) (data not shown). The maximum non-toxic concentrations of each of organotin compounds were 0.1 μM tributyltin chloride, 0.1 μM dibutyltin diacetate, 0.1 μM dibutyltin dichloride, 1 μM dibutyltin dilaurate, or 0.1 μM dibutyltin maleate. After 14 days, cells were stained with Oil Red O (Sigma–Aldrich), as previously described (Janderová et al., 2003).

For gene expression analysis by real-time PCR, preadipocytes were differentiated and exposed to vehicle (DMSO), 0.1 μM rosiglitazone, and to the concentrations that induced the highest lipid accumulation determined by Oil Red O staining, which were 0.1 μM tributyltin chloride, 0.1 μM dibutyltin diacetate, 0.1 μM dibutyltin dichloride, 1 μM dibutyltin dilaurate, or 0.1 μM dibutyltin maleate. To assess expression of adipogenesis-related genes (*Fabp4*, *Adipoq*, and *Glut4*), preadipocytes were exposed to vehicle, rosiglitazone or dibutyltin for three days in the presence or absence of a potent and selective PPARγ antagonist, T0070907 (1 μM). To evaluate the effect of dibutyltins on inflammation-related genes (*Lgals9*, *Fn1*, *Dcn*, *Vcam1*, and *S100a8*), preadipocytes were differentiated and exposed to the different compounds for 14 days.

### Western Blotting Analysis

After 14 days of differentiation, 12.5 μg of total protein extracted from adipocytes 3T3-L1 was separated by SDS-PAGE, transferred to a PVDF membrane in a semi-dry Transfer Cell (Bio-RAD), incubated with FABP4 and GAPDH antibodies (Abcam) and was then immunodetected by chemiluminescence (ECL prime kit, GE Healthcare Life Sciences). The immunoblots were visualized with peroxidase-conjugated secondary antibodies mouse anti-goat IgG-horseradish peroxidase conjugate (HRP) antibody (Santa Cruz Biotechnology).

### Macrophage LPS Stimulation Assay

Murine RAW 264.7 macrophages were cultured in 12-well plates (15 × 10^4^ cells/well) and after 24 h exposed to vehicle (DMSO), 10 μM rosiglitazone, 10 μM 9-*cis*-retinoic acid or with the maximum non-toxic concentration of organotin compounds determined in cell viability assays, performed as previously described (Mosmann, 1983) (data not shown) which were 0.1 μM for tributyltin chloride, 1 μM for dibutyltin diacetate, 0.1 μM for dibutyltin dichloride, 1 μM for dibutyltin dilaurate, and 0.1 μM for dibutyltin maleate. After 4 h, 100 ng/mL LPS (Sigma–Aldrich) was added, and after 24 h cells were collected for analysis of *TNFα* gene mRNA expression.

### Real-Time PCR

Total RNA from 3T3-L1 and RAW 264.7 cells was isolated by using TRIzol reagent (Invitrogen), following the manufacturer’s protocol, and treated with DNase I (Sigma–Aldrich). RNA (5 ng) was then reverse transcribed, and quantitative real-time PCR was carried out using Power SYBR^®^ Green RNA-to-CT 1-Step kit (Applied Biosystems) in an AB7500 PCR machine (Applied Biosystems). Normalized *Cp* values (Critical point; *Cp*_Target_–*Cp_Gapdh_*) of at least six biological replicates were analyzed by ANOVA followed by *t*-tests comparing DMSO (vehicle) vs. compounds, with the compound’s effect determined to be significant with a Bonferroni Corrected *p*-value < 0.05 (GraphPad Software, version 5.01). Primer sequences were as follows: *Fabp4*, sense 5′-CCATCTAGGGTTATGCTCTCTCA-3′, antisense 5′-ACACCGAGATTTCCTTCAAACTG-3′; *Adipoq*, sense 5′-GCACTGGCAAGTTCTACTGCAA-3′, anti-sense 5′-GTAGGTGAAGAGAACGGCCTTGT-3′; *Glut4*, sense5′-TCATTGTCGGCATGGGTTT-3′, antisense 5′-CGGCAAATAGAAGGAAGACGTA-3′; *Lgals9*, sense 5′-TACCAACACCGCGTACCCTA-3′, antisense 5′-GCTGCAGAGTTCTGGAAGGTG-3′; *Fn1*, sense 5′-CTGCGCTCCATTCCACCTTA-3′, antisense 5′-GGTCGTACACCCAGCTTGAA-3′; *S100a8*, sense5′-TTCAAGACATCGTTTGAAAGGAAA-3′, antisense 5′-AGGTTGCTCAAGGCCTTCTC-3′; *Dcn*, sense 5′-TCACAGAAGCGGTAACGAGC-3′, antisense 5′-TCATGTATTTTCACGACCTTCTGA-3′; *Vcam1*, sense 5′-TGGAGGTCTACTCATTCCCTGA-3′, antisense 5′-GACAGGTCTCCCATGCACAA-3′; *TNFα*, sense 5′-CCCTCACACTCAGATCATCTTCT-3′, antisense5′-GCTACGACGTGGGCTACAG-3′; *Gapdh*, sense 5′-AAGGGCTCATGACCACAGTC-3′, antisense 5′-CAGGGATGATGTTCTGGGCA-3′.

## Results

### Dibutyltins Are PPARγ and RXRα Agonists

**FIGURE 1:**
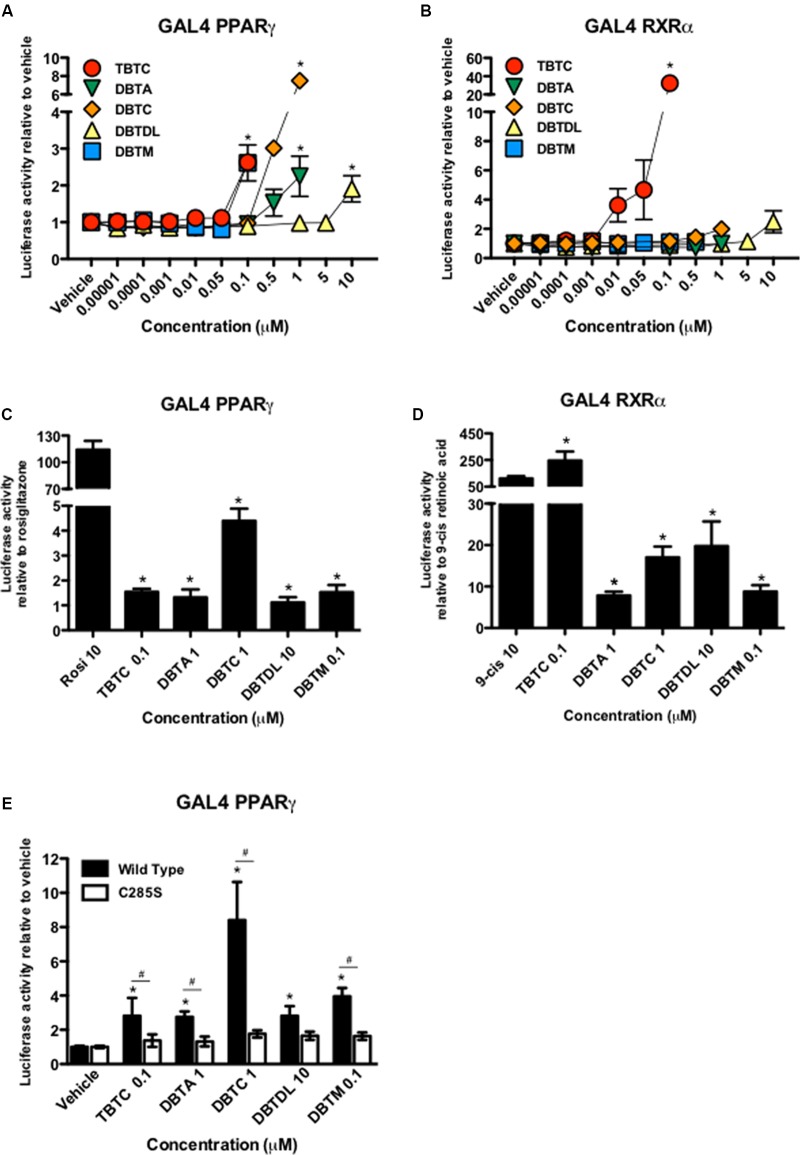
Dibutyltins are partial PPARγ and RXRα agonists, and cysteine 285 is important in PPARγ transcriptional activity by these compounds. HeLa cells were co-transfected with plasmids containing cDNA of GAL4-PPARγ **(A,C)**, GAL4-RXRα **(B,D)** or GAL4-PPARγ C285S **(E)** and the responsive element GAL4 fused to luciferase reporter gene. **(A,B)** Cells were exposed to vehicle (DMSO), tributyltin chloride (TBTC), dibutyltin diacetate (DBTA), dibutyltin dichloride (DBTC), dibutyltin dilaurate (DBTDL), or dibutyltin maleate (DBTM) from 0.00001 μM to the maximum non-toxic concentration. ^∗^*p* ≤ 0.01 (compared to vehicle). **(C,D)** Cells were exposed to rosiglitazone (Rosi) 10 μM, or 9-*cis*-retinoic acid (9-*cis*) 10 μM, or the maximum non-toxic concentrations of each of organotin compounds. ^∗^*p* ≤ 0.0001 compared to rosiglitazone (Rosi) or 9-*cis*-retinoic acid (9-*cis*). **(E)** Cells exposed to vehicle or maximum non-toxic concentrations of each of organotin compounds. ^∗^*p* ≤ 0.0001 (compared to vehicle) and ^#^*p* ≤ 0.01 compared wild type *versus* C285S. Data are presented as mean (SD) of three independent experiments conducted in triplicate.

As previously described, tributyltin chloride induced PPARγ and RXRα transcriptional activation, displaying a partial agonist effect of PPARγ and a total agonist of RXRα, compared to the full agonists rosiglitazone and 9-*cis*-retinoic acid, respectively (**Figures 1A–D**). Similarly to tributyltin chloride (2.63 ± 0.21-fold), the organotins dibutyltin diacetate (2.25 ± 0.55-fold), dibutyltin dichloride (7.50 ± 0.84-fold), dibutyltin dilaurate (1.91 ± 0.36-fold), and dibutyltin maleate (2.61 ± 0.49-fold) activated PPARγ as partial agonists (**Figures 1A,C**). Dibutyltin dichloride and dibutyltin dilaurate also displayed partial agonism on RXRα (1.99 ± 0.41, and 2.49 ± 0.75-fold, respectively), albeit with reduced efficacy when compared with tributyltin chloride (32.47 ± 7.07-fold) (**Figures 1B,D**).

Tributyltin chloride, dibutyltin dilaurate and dibutyltin maleate only activated PPARγ at their highest concentrations, whereas dibutyltin diacetate, and dibutyltin dichloride were active at the two highest concentrations tested (**Figure 1A**). Dibutyltin dichloride and dibutyltin dilaurate only activated RXRα at their highest concentrations (**Figure 1B**).

Structural studies have shown that tributyltin chloride, like some endogenous lipids (Itoh et al., 2008; Waku et al., 2009), binds to PPARγ through ionic interactions between the tin atom and the sulfur atom of Cys285 in the ligand binding pocket (Harada et al., 2015). Therefore, we replaced Cys285 by serine (C285S) in PPARγ LBD and examined the effect of several organotins on the transcriptional activity of this mutant. As shown in **Figure 1E**, C285S mutation abolished the induction of PPARγ transcriptional activity by all compounds tested, including dibutyltins.

### Dibutyltins Induce Adipogenesis in a PPARγ-Dependent Manner

**FIGURE 2:**
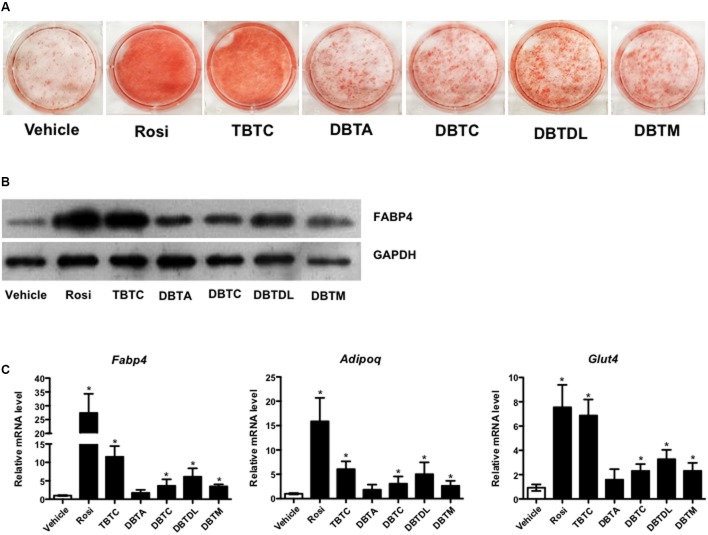
Dibutyltins promote adipogenesis in 3T3-L1. Cells were induced to differentiate with insulin, and exposed to vehicle (DMSO), Rosi 0.1 μM, TBTC 0.1 μM, DBTA 0.1 μM, DBTC 0.1 μM, DBTDL 1 μM, or DBTM 0.1 μM. **(A)** After 14 days the cells were fixed, stained with oil red O, and photo documented. **(B)** Western blot to evaluate the expression of FABP4 protein after 14 days of differentiation. **(C)** Real-time quantitative PCR to evaluate the expression of *Fabp4*, *Adipoq*, and *Glut4* genes after 3 days of differentiation. Data are presented as mean (SD) of three independent experiments conducted in triplicate and expressed as activation relative to transcript levels in vehicle samples (DMSO). ^∗^*p* ≤ 0.05.

All dibutyltins tested increased intracellular lipid accumulation in the 3T3-L1 cell line, with dibutyltin dilaurate having the most pronounced effect (**Figure 2A**). The adipogenic action occurred in a dose dependent-manner (not shown), and was confirmed by *Fabp4* mRNA and protein expression (**Figures 2B,C**). Moreover, a significant increase in the expression of *Adipoq* and *Glut4* was detected in preadipocytes induced to differentiate in the presence of all tested organotin compounds, except dibutyltin diacetate (**Figure 2C**).

**FIGURE 3:**
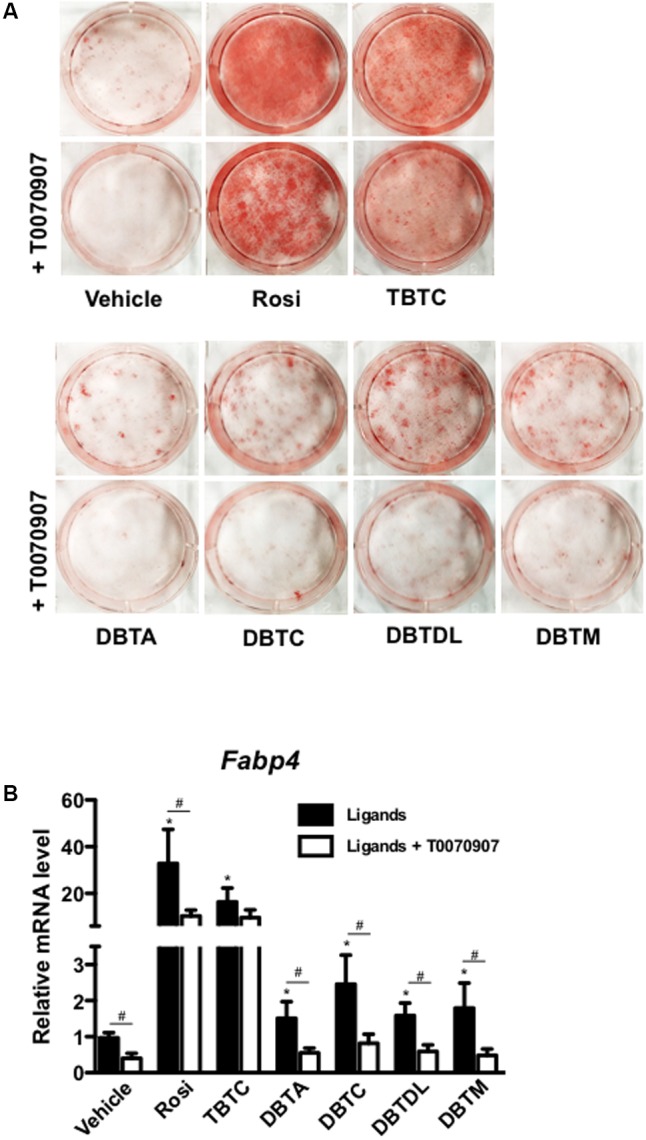
Dibutyltins’s adipogenic effect is PPARγ dependent. **(A)** 3T3-L1 cells were induced to differentiate with insulin, and exposed to vehicle (DMSO), Rosi 0.1 μM, TBTC 0.1 μM, DBTA 0.1 μM, DBTC 0.1 μM, DBTDL 1μM, or DBTM 0.1 μM in the presence or absence of a PPARγ specific antagonist, T0070907 1 μM. After 14 days of differentiation, the cells were fixed and stained with oil red O, and photo documented. **(B)** Cells were differentiated for 3 days and collected for gene expression evaluation of *Fabp4* by real-time quantitative PCR. Data are presented as mean (SD) of three independent experiments conducted in triplicate and expressed as activation relative to transcript levels in vehicle samples (DMSO). ^∗^*p* ≤ 0.01 (compared to vehicle in the absence of T0070907), and ^#^*p* ≤ 0.001 (compared to the same ligand in the presence and the absence of T0070907).

We then tested the PPARγ-dependence of dibutyltins adipogenic action by inducing 3T3-L1 cells to differentiate in the absence or presence of the selective PPARγ antagonist T0070907. Exposing 3T3-L1 cells to dibutyltins in the presence of T0070907 decreased intracellular lipid accumulation and expression of the *Fabp4* gene, suggesting that the adipogenic effect of organotin compounds is mediated by PPARγ (**Figures 3A,B**). Notably, T0070907 completely impaired adipogenesis induced by dibutyltins, but not by rosiglitazone or tributyltin chloride (**Figure 3A**). Moreover, T0070907 strongly reduced *Fabp4* gene expression induced by dibutyltins but not by rosiglitazone and tributyltin chloride (**Figure 3B**).

### Dibutyltins Display a Partial Anti-inflammatory Effect in Cell Culture

**FIGURE 4:**
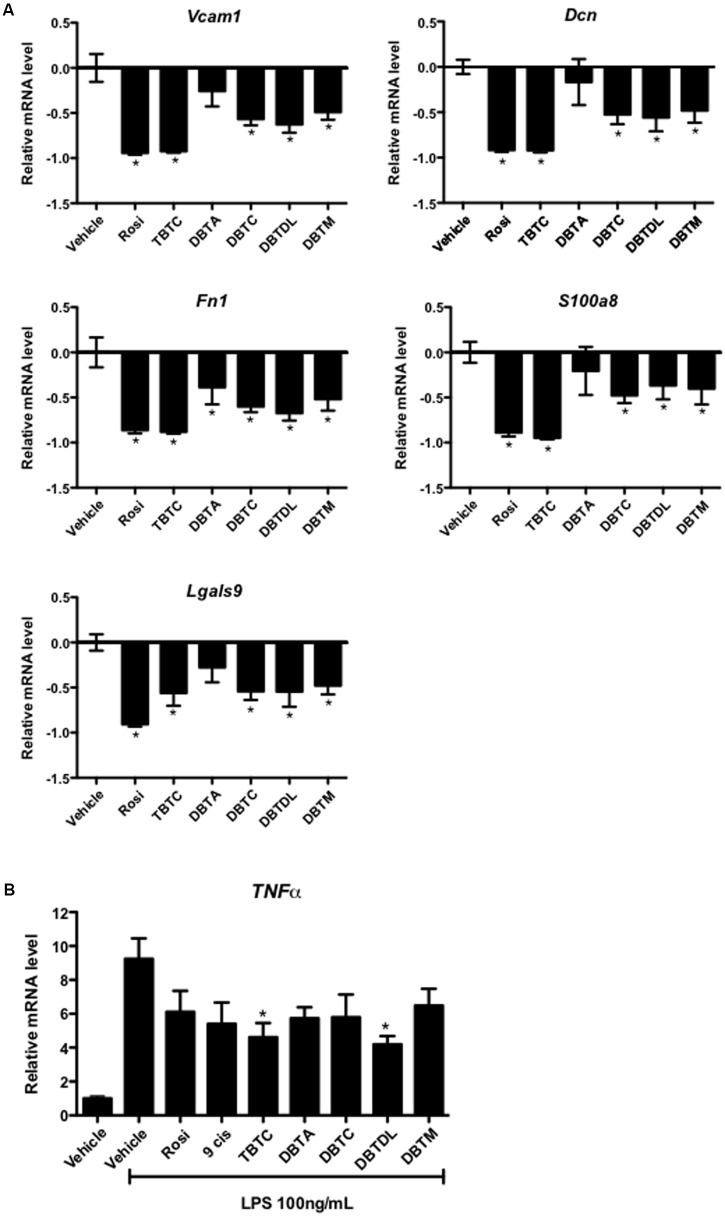
Dibutyltins inhibited the expression of inflammatory genes. **(A)** 3T3-L1 cells were differentiated with insulin, and exposed to vehicle (DMSO), Rosi 0.1 μM, TBTC 0.1 μM, DBTA 0.1 μM, DBTC 0.1 μM, DBTDL 1 μM, or DBTM 0.1 μM. After 14 days, the cells were collected for gene expression evaluation of *Vcam1*, *Dcn*, *Fn1*, *S100a8*, and *Lgals9* by real-time quantitative PCR. Data are expressed as fold activation relative to transcript levels in vehicle samples (DMSO). ^∗^*p* ≤ 0.01 (compared to vehicle samples). **(B)** Raw 264.7 Cells were exposed to vehicle (DMSO), Rosi 10 μM, 9-*cis*-retinoic acid (9-*cis*) 10 μM, TBTC 0.1 μM, DBTA 1 μM, DBTC 0.1 μM, DBTDL 1 μM, or DBTM 0.1 μM. After 4 h, LPS 100 ng/mL was added and maintained for an additional 24 h. After that, the cells were collected for evaluation of gene expression of *TNFα* by real-time quantitative PCR. Data are presented as mean (SD) of three independent experiments conducted in triplicate and expressed as activation relative to transcript levels in vehicle samples (DMSO). ^∗^*p* ≤ 0.01 (compared to vehicle + LPS samples).

In 3T3-L1 adipocytes, all organotins studied, but not dibutyltin diacetate repressed the expression inflammatory response-related genes (*Lgals9*, *Fn1*, *Dcn*, *Vcam1*, and *S100a8*). The inhibitory effect of tributyltin chloride was similar to that of rosiglitazone, whereas dibutyltin dichloride, dibutyltin dilaurate, and dibutyltin maleate had only moderate effects on these genes (**Figure 4A**). In RAW 264.7 cells, all the ligands reduced LPS-stimulated *TNFα* expression, although only tributyltin chloride and dibutyltin dilaurate exhibited significant reduction (**Figure 4B**).

## Discussion

The Environmental Protection Agency (EPA) defines endocrine disruptors as “chemicals that may interfere with the endocrine system” (United States Environmental Protection Agency [EPA], 2015). Endocrine disruptors comprise a heterogeneous group of compounds (Kabir et al., 2015) that includes organotins (Nakanishi, 2008). The best-described organotin is tributyltin chloride, banned as antifouling paint in 2008 because of the risks to marine ecosystems (Gipperth, 2009). The most researched dibutyltin is dibutyltin dichloride, which, besides being directly produced as dibutyltin, is also a metabolite of tributyltin chloride (Ohhira et al., 2003). Like tributyltin chloride, dibutyltin dichloride is hepatotoxic (Ueno et al., 2003), neurotoxic (Chantong et al., 2014), and immunotoxic (Dudimah et al., 2007a). However, there are 17 other dibutyltin compounds registered in ECHA besides dibutyltin dichloride, of which the European Economic Area (EEA) manufactures or imports 10 to 100 tons per year (European Chemicals Agency [ECHA], 2016). However, unlike tributyltin chloride and dibutyltin dichloride, the biological effects of other dibutyltin compounds have been addressed in only a few studies, raising questions about how they affect the environment and humans.

The adipogenic effect of tributyltin chloride and its dependence upon PPARγ/RXRα has been previously described (Kirchner et al., 2010; Li et al., 2011). However, to our knowledge, no study has been reported to date that the dibutyltin compounds acts as a partial PPARγ and RXRα agonist with significant adipogenic potential. Consequently, to gather information on dibutyltin compounds, we analyzed the effects of four of these endocrine disruptors on PPARγ and RXRα transcriptional activity and PPARγ associated biological effects (adipogenesis and inflammatory response).

Dibutyltin diacetate, dibutyltin dilaurate, and dibutyltin maleate induced the transcriptional activity of PPARγ in a magnitude comparable to that of tributyltin chloride, whereas dibutyltin dichloride was about three times more efficient than tributyltin chloride. Tributyltin chloride’s partial agonist activity on PPARγ was previously described (Grün et al., 2006; le Maire et al., 2009), but the activity of dibutyltins on this nuclear receptor has not been reported. Hiromori et al. (2009) investigated the effect of dibutyltin dichloride on the transcriptional activity of PPARγ in JEG-3 cells and found no significant effect. Differences related to the cell line used in this study, in addition to the higher concentration of dibutyltin dichloride tested (10 times higher than that used by Hiromori et al., 2009), might explain the distinct results.

Tributyltin chloride activated RXRα as a full agonist, while dibutyltin dichloride and dibutyltin dilaurate functioned as partial agonists. The total agonism of tributyltin chloride on RXRα was previously reported (Grün et al., 2006; le Maire et al., 2009), as was the partial activation of this receptor by dibutyltin dichloride (Grün et al., 2006). On the other hand, this is the first report that dibutyltin dilaurate is a partial RXRα agonist.

Previous studies demonstrated that tributyltin chloride directly interacts with RXRα and PPARγ LBDs (Grün et al., 2006). The crystallographic structure of tributyltin chloride bound to RXRα was solved in 2009 (le Maire et al., 2009). More recently, the structure of tributyltin chloride bound to PPARγ was also determined, and it was shown that tributyltin chloride binds to PPARγ LBD by a non-covalent, ionic bond between the tin atom and Cys285 (Harada et al., 2015). We found that substitution of cysteine 285 for serine abolished the transcriptional activity of PPARγ induced by dibutyltin. This result suggests that similarly to tributyltin chloride (Harada et al., 2015), there may be an interaction between the cysteine residue and tin atom in the dibutyltin compounds that is essential for PPARγ activation by these compounds. It is hence plausible that dibutyltins bind to PPARγ binding pocket similarly to tributyltin chloride. Interesting, this residue was shown to be essential for PPARγ activation by certain endogenous ligands (Waku et al., 2009).

Our findings also indicated the adipogenic action of dibutyltin compounds in 3T3-L1 cells, although less pronounced than that of tributyltin chloride. The adipogenic effect of tributyltin chloride was formerly demonstrated in 3T3-L1 cells (Grün et al., 2006), and in primary human (Kirchner et al., 2010) and murine (Kirchner et al., 2010; Biemann et al., 2012) mesenchymal cells.

Surprisingly, the adipogenic effect of rosiglitazone or tributyltin chloride was not inhibited by the specific PPARγ antagonist T0070907, although this compound completely impaired adipogenesis induced by dibutyltins. Previous results from Kirchner and colleagues also indicated that the inhibitory effect of T0070907 on rosiglitazone-induced adipogenesis was not observed in the presence of ten-fold higher concentration of rosiglitazone when compared to T0070907 (Kirchner et al., 2010). Nonetheless, we found the same effect even when T0070907 was at a concentration ten-fold higher than rosiglitazone. The interpretation of this disagreement is not clear, but differences in cell type or the presence of a PPARγ alternative binding site that is not blocked by T0070907 (Hughes et al., 2014) need further investigation. Modulation of PPARγ target genes by tributyltin chloride was previously shown (Kirchner et al., 2010), and the binding of this compound to PPARγ ligand binding domain was detailed by crystallographic structure (Harada et al., 2015). However, it is not possible to rule out that the mechanism by which T0070907 did not disrupt tributyltin chloride-induced adipogenesis is mediated by a PPARγ independent action. T0070907 recruits the NCoR and SMRT co-repressors to the PPARγ/RXRα heterodimer, preventing its activation (Lee et al., 2002). RXRα agonists inhibit this recruitment so that T0070907 effects can be diminished by RXRα ligands, depending on their concentration and availability (Lee et al., 2002). Accordingly, it is conceivable that tributyltin chloride induced adipocyte differentiation in the presence of T0070907 because it is a full RXRα agonist and displaced co-repressors from the PPARγ/RXRα heterodimer complex. On the other hand, although dibutyltin dilaurate is a partial RXR agonist, it was also unable to induce adipocyte differentiation in the presence of T0070907. This result may be explained by the less pronounced agonist effect of dibutyltin dilaurate on RXRα, which was ten-fold lower than that of tributyltin chloride. Taken together, these results suggested that adipogenic effect of tributyltin chloride depends on PPARγ and RXRα activation, despite the dependency of dibutyltins’ effect upon PPARγ seemed more pronounced.

The effect of all tested compounds on the expression of adipogenesis-related genes, *Fabp4*, *Adipoq*, and *Glut4*, was consistent with their action on intracellular lipid accumulation. Tributyltin chloride strongly induced the expression of these genes, while dibutyltins caused only moderate induction, with dibutyltin diacetate showing no significant effect. The marked induction of *Fabp4* expression by tributyltin chloride in 3T3-L1 cells was consistent with previous reports (Grün et al., 2006). The effect of this compound on adipogenesis-related genes was previously demonstrated in human and murine mesenchymal cells (Kirchner et al., 2010) and in mice exposed to tributyltin chloride during congenital life (Grün et al., 2006). The effect of tributyltin chloride on induction of *Adipoq* expression was also previously described in 3T3-L1 cells, although this result was observed when 3T3-L1 cells were exposed to isobutylmethylxanthine, but not with insulin (Regnier et al., 2015).

It should be pointed that the concentrations of tributyltin chloride and dibutyltin compounds required for induction of adipogenesis did not parallel those required for PPARγ activation in reporter-gene assays. This difference may be explained in the different cellular context in each assay, including different cell strains used (human cells in transactivation assays and murine cells in adipogenesis assays). Notwithstanding, the dependency of tributyltin chloride and dibutyltin compounds-induced adipogenesis upon PPARγ activation was shown by the inhibitory effects of the PPARγ antagonist T0070907.

PPARγ is the key regulator of adipogenesis, and its activation is also associated with anti-inflammatory effects (Lehrke and Lazar, 2005). Taking into account the partial agonism to this receptor by tributyltin chloride and by the dibutyltins studied herein, we investigated the effect of these compounds on the expression of inflammatory genes in two murine cell models, 3T3-L1 adipocytes and RAW 264.7 macrophages. The latter cells are one of the most well-established models to study the molecular events involved in the inflammatory response (Ricote and Glass, 2007).

In 3T3-L1 adipocytes, we evaluated the effect of organotins on the expression of inflammatory response-related genes such as *Lgals9*, *Fn1*, *Dcn*, *Vcam1* and *S100a8*, which are accordingly related to obesity and insulin resistance (Andersen et al., 1987; Osborn et al., 1989; Daquinag et al., 2011; Xue et al., 2011; Sekimoto et al., 2012; Kurose et al., 2013). These genes were chosen from previous data (Milton et al., 2015), indicating their repression by rosiglitazone (a complete PPARγ agonist) and GQ-16 (a partial PPARγ agonist) using microarray assays. In this experimental model, tributyltin chloride showed a strong repressor effect on those genes, similar to rosiglitazone, whereas dibutyltin dichloride, dibutyltin dilaurate, and dibutyltin maleate showed a less pronounced, but significant effect, similar to PPARγ GQ-16 and MLR-24 partial agonists (Milton et al., 2015). In contrast, in the LPS-stimulated RAW 264.7 macrophages, only tributyltin chloride and dibutyltin dilaurate significantly suppressed *TNFα* expression. The effect of organotins on inflammatory response was investigated previously but in a different context. Exposure to tributyltin chloride reduced the anti-inflammatory activity (Aluoch et al., 2006; Dudimah et al., 2007b) and induced apoptosis of immune cells (Lavastre and Girard, 2002).

Our results confirm that tributyltin chloride displays anti-inflammatory effect as well as rosiglitazone. Nevertheless, in RAW 264.7 cells, tributyltin chloride and dibutyltin dilaurate impaired LPS-stimulated *TNFα* expression more strongly than rosiglitazone. Since tributyltin chloride, but not dibutyltins, is as strong RXRα agonist, and C285S mutation in PPARγ LBD abrogated dibutyltins transcription activity, we suppose that dibutyltins anti-inflammatory effect was predominantly PPARγ-dependent (**Figure 4B**). Regardless, the effect of dibutyltins on inflammatory response-related genes was similar to previous results reported by Nakamuta et al., who observed a reduction in *TNFα* production in LPS-stimulated RAW 264.7 cells exposed to BADGE (an endocrine disruptor that operates as a PPARγ agonist in this cell line) (Nakamuta et al., 2002).

The translation of these findings on organotin compounds into metabolic impacts *in vivo* may be difficult to predict. Nevertheless, few studies have addressed this issue. Data from Zuo et al. (2009) indicated that tributyltin chloride induced hepatic steatosis, augmented weight and visceral adipose tissue mass, in addition to hyperinsulinemia (Zuo et al., 2009). Additionally, Penza et al. (2011) showed that mice exposed to tributyltin chloride increased adipose tissue mass without the changes in body weight or glucose tolerance (Penza et al., 2011). Obesity and hepatic steatosis in rodents are consistent with the agonist activity of tributyltin chloride on PPARγ (Edvardsson et al., 1999; García-Ruiz et al., 2007; Rull et al., 2014). However, increased visceral adipose mass and hyperinsulinemia are not expected effects of PPARγ and RXRα activation. Concerning the effects of human exposure to these compounds, there are few reports in the literature. Kannan et al. (1999) measured the concentrations of tin compounds in the blood of 32 volunteers, 17 males and 15 females. They have reported the presence of monobutyltin, dibutyltin, and tributyltin in 53, 81, and 70% of the samples evaluated, respectively. The mean overall concentration of dibutyltin and tributyltin was 4.94 and 8.18 ng/mL, respectively (Kannan et al., 1999).

Our findings indicate that the organotins dibutyltin diacetate, dibutyltin dichloride, dibutyltin dilaurate, and dibutyltin maleate are partial PPARγ agonists and that dibutyltin dichloride and dibutyltin dilaurate are also partial RXRα agonists. To our knowledge, these effects had not been addressed before. Moreover, tributyltin chloride and dibutyltin compounds repressed inflammatory response genes and induced adipogenesis. Although the translation of cell-based results assays into *in vivo* effects is not entirely known, the current findings open new avenues to understand how organotin compounds may affect the human health. Nevertheless, further studies are necessary to address their effects in different periods of life and to elucidate the complex actions of organostannic compounds in whole-body context.

## Author Contributions

FN and AA conceived study; FN, AA, and FM contributed to the experimental design; FM, SS, and DP conduced the transactivation assay; FM, SS, ML, and GP performed the adipocyte differentiation assay; ML, MB, and PM performed the western blotting; AM and FM conduced the macrophage stimulation assay; FM, ML, MC, CdL, and AM contributed with PCR; FM, ML, and PM wrote the manuscript; FN, AA, MB, and AM reviewed the manuscript, FM and ML edited the manuscript.

## Conflict of Interest Statement

The authors declare that the research was conducted in the absence of any commercial or financial relationships that could be construed as a potential conflict of interest.
